# Islamist insurgency and the war against polio: a cross-national analysis of the political determinants of polio

**DOI:** 10.1186/s12992-015-0123-y

**Published:** 2015-09-30

**Authors:** Jonathan Kennedy, Martin McKee, Lawrence King

**Affiliations:** Department of Political Science, School of Public Policy, University College London, 29-31 Tavistock Square, London, WC1H 9QU UK; London School of Hygiene and Tropical Medicine, European Centre on Health of Societies in Transition, 15-17 Tavistock Place, London, WC1H 9SH UK; Department of Sociology, University of Cambridge, Free School Lane, Cambridge, CB2 1TN UK

**Keywords:** Polio eradication, Vaccination programmes, Civil War, Insurgency, Islamism, Taliban, Boko Haram, Al-Qaeda, Osama bin Laden, Political determinants of health

## Abstract

**Background:**

There is widespread agreement that civil war obstructs efforts to eradicate polio. It is suggested that Islamist insurgents have a particularly negative effect on vaccination programmes, but this claim is controversial.

**Methods:**

We analyse cross-national data for the period 2003–14 using negative binomial regressions to investigate the relationship between Islamist and non-Islamist insurgency and the global distribution of polio. The dependent variable is the annual number of polio cases in a country according to the WHO. Insurgency is operationalized as armed conflict between the state and an insurgent organization resulting in ≥25 battle deaths per year according to the Uppsala Conflict Data Programme. Insurgencies are divided into Islamist and non-Islamist insurgencies. We control for other possible explanatory variables.

**Results:**

Islamist insurgency did not have a significant positive relationship with polio throughout the whole period. But in the past few years – since the assassination of Osama bin Laden in 2011– Islamist insurgency has had a strong effect on where polio cases occur. The evidence for a relationship between non-Islamist insurgency and polio is less compelling and where there is a relationship it is either spurious or driven by ecological fallacy.

**Conclusions:**

Only particular forms of internal armed conflict – those prosecuted by Islamist insurgents – explain the current global distribution of polio. The variation over time in the relationship between Islamist insurgency and polio suggests that Islamist insurgent’s hostility to polio vaccinations programmes is not the result of their theology, as the core tenets of Islam have not changed over the period of the study. Rather, our analysis indicates that it is a plausibly a reaction to the counterinsurgency strategies used against Islamist insurgents. The assassination of Osama bin Laden and the use of drone strikes seemingly vindicated Islamist insurgents’ suspicions that immunization drives are a cover for espionage activities.

**Electronic supplementary material:**

The online version of this article (doi:10.1186/s12992-015-0123-y) contains supplementary material, which is available to authorized users.

## Background

In 1988 the Global Poliomyelitis Eradication Initiative (GPEI) launched a global campaign to eradicate poliovirus by the turn of the century through a programme of mass immunization. Polio has not been eradicated, but the GPEI has achieved remarkable results: in 1988 there were more than 350,000 polio cases in over 125 countries, while in 2014 there were 359 cases of wild polio in ten countries [1–3]. Poliovirus has remained endemic in three countries: Nigeria, Pakistan, and Afghanistan. In the past couple of years polio has spread from endemic countries to cause outbreaks in previously polio free countries: from Nigeria to other countries in West Africa and the Horn of Africa – there were 199 polio cases in Somalia in 2013–14 (the first since 2007); and from Pakistan/Afghanistan to the Middle East – there were 38 cases in Syria and Iraq in 2013–14 (the first since 1999 and 2000 respectively). In May 2014 the WHO declared the renewed spread of poliovirus to be an “extraordinary event” and a “public health emergency of international concern” [4].

Empirical experience demonstrates that eradication is possible. India was until recently the largest endemic reservoir of polio – in 2009 it accounted for almost half of the world’s polio cases – and was considered to be the most difficult challenge for eradication [3, 5]. But India has not had a polio case since 2011 and in 2014 it was declared polio free. This success was the result of two factors. First, new vaccines have been developed against specific strains of the virus. These are particularly effective in areas where sanitation is poor because bacteria that infect the gut interfere with the body’s ability to mount an effective immune response to older vaccines. Second, a concerted effort by the Indian government, in collaboration with the GPEI and other organizations, deployed a large number of workers (more than 250,000 in the state of Uttar Pradesh alone), and paid particular attention to vaccinating children from migratory populations and in dangerous and remote areas [5]. The eradication of polio in India removed any doubts regarding the feasibility of polio eradication. As Bruce Aylward, WHO Assistant Director-General for Polio, pointed out, “Now that India has become polio-free, we have crossed… from our primary barrier being technological or biological feasibility to one of political and societal will” [6].

The technical and biological factors that inhibit polio eradication are well understood. The organisational factors are also understood [7]. The proximate cause of the persistence of polio in some areas and new outbreaks in previously polio free areas is that too few children are vaccinated to stop the spread of poliovirus [8, 9]. Yet the underlying political and societal factors that inhibit the effective implementation of vaccination programmes have attracted little systematic analysis and do not even feature in assessments of risks of outbreaks [10]. There is widespread agreement that civil war is associated with disease in general [11] and barriers to polio eradication in particular [1, 6, 12, 13]. Insurgency diverts resources away from healthcare and public health programmes, disrupts healthcare infrastructure and the organisation of vaccination programmes, and leads to forced migration, which spreads infectious disease and makes populations hard to reach. Reports in newspapers and medical journals have suggested that Islamist insurgents have a particularly negative effect on polio because they deliberately undermine the efficacy of polio immunization campaigns by spreading misinformation that they are a conspiracy to sterilize Muslim populations, which increases the likelihood that parents will refuse vaccinations [8], and carrying out targeted violence and boycotts – often legitimised by these rumours – that reduce the ability and willingness of polio workers to operate in particular areas [12]. Some accounts argue that this reflects Islamists’ adherence to Islam and rejection of non-Islamic influences, which makes them deeply antagonistic towards non-Muslims and the West in particular [14]. More nuanced interpretations suggest that Islamist insurgents’ animus towards eradication programs must be understood in the context of their interaction with domestic political rivals and international actors [15, 16]. Islamic scholars note that there is no religious basis for opposition to polio immunisation and suggest that the primary reason for failure of eradication is the presence of conflict [17].

The first major conflict between Islamists – albeit non-violent Islamists – and polio campaigns occurred in 2003, when the leaders of several northern-Nigerian states banned vaccination programmes following rumours that they were a Western conspiracy to render Muslim children infertile [9, 16, 18]. The boycott lasted a year and was a major setback for polio eradication. It resulted in a global polio outbreak that affected 20 countries, accounted for 80 % of the world’s polio cases at the time, and cost more than US$500 million to control [18]. In Pakistan, resistance to polio campaigns began a few years later: in 2007 militants banned vaccination programmes in the North West Frontier Province due to similar fears [19]. The boycott was accompanied by targeted violence against polio workers – most notably the assassination of the head of the government’s vaccination campaign in Bajaur Agency in 2007. Some observers argue that Islamist insurgents in Pakistan have become increasingly hostile to polio vaccinations in the past couple of years. These accounts stress the CIA’s use of a fake hepatitis immunisation programme to collect DNA from Osama bin Laden’s family members before his assassination in 2011. This seemingly vindicated insurgents’ suspicions that immunization drives are a cover for espionage activities [6, 9, 20]. In addition, the increased use of drone strikes in northwest Pakistan by the United States is said to have amplified Islamist insurgents’ enmity to polio vaccination campaigns because the insurgents suspect that polio workers were carrying out surveillance in order to identify targets for drone strikes [2, 6, 21, 22]. As a result an influential leader of the Pakistani Taliban in North Waziristan banned polio vaccination programmes in areas under his control in summer 2012 [20, 21]. This is said to have led to a steep increase in the number of polio cases in the area [20, 21]. It should be noted, however, that other influential Islamist clerics in Pakistan opposed the ban, issuing Fatwas that encouraging parents to immunize their children against polio and other diseases [23]. In addition, Boko Haram has reportedly carried out several similar attacks on polio workers in northern Nigeria [9, 24]. There have also been alleged attacks in Afghanistan, although these are much less frequent [25]. It should be noted, however, that when the Taliban were in power between 1995 and 2001 they fully supported the GPEI. They continue to support polio campaigns but the diffusion of ideas from Pakistan means that some insurgents are hostile to vaccination programmes and many parents refuse to vaccinate their children [9, 26, 27].

Based on the analysis outlined above we generate three testable hypotheses. The first relates to the widely held conviction that civil war in general increases the likelihood that a country will be affected by polio. It is argued that the violence and disruption of armed conflict undermines the ability of polio workers to carry out mass vaccination programmes, as well as causing a more general public health crisis.

Hypothesis 1: Countries affected by non-Islamist insurgency will have a higher number of polio cases.

The second hypothesis considers the more contentious argument that Islamist insurgency in particular increases the likelihood that a country will be affected by polio. It is argued that Islamist insurgents deliberately undermine the effectiveness of polio immunization campaigns by spreading misinformation and carrying out targeted violence and boycotts.

Hypothesis 2: Countries affected by Islamist insurgency will have a higher number of polio cases.

Thirdly, some observers argue that Islamist insurgents’ animosity towards polio vaccination programmes is the logical result of Islamic theology. If this is the case we would expect the hostility to be more or less constant to reflect the fact that the theological tenets of Islam have not changed over the past decade or so. Alternatively, others stress the role of political dynamics. It seems apparent that some Islamist insurgents have come to realize that interrupting polio campaigns is a useful strategy because it generates international attention for the insurgents and enables them to force concessions from their opponents. Moreover, it is argued that Islamist insurgents’ enmity towards polio vaccination programmes has intensified in recent years in response to the counterinsurgency strategies used against them. The increased use of drone attacks and the CIA’s use of a fake immunisation program in the assassination of Osama bin Laden seemingly vindicated Islamist insurgents’ suspicions that immunization drives are a cover for espionage activities. This is said to have seriously compromised the GPEI’s activities.

Hypothesis 3: The effect of Islamist insurgency on the incidence of polio will be stronger after the assassination of Osama bin Laden in 2011.

## Methods

We analyse cross-national data to test the hypotheses. This paper is, to the best of our knowledge, the first study to systematically analyse the relationship between insurgency and the persistence of polio with quantitative data. Until now our understanding of this topic is informed by qualitative data. This is potentially problematic for two reasons, one general, the other specific to Islamist insurgency. First, it is plausible that many of the conditions that increase the probability of insurgency also make a country more likely to experience a polio outbreak. In other words there may be spurious causality. Insurgency and polio are both more likely to occur in less-developed societies because the opportunity cost of being involved in insurgency is likely to be lower [28] and the general health of the population tends to worse and the opportunity cost of being involved in insurgency is likely to be lower [29]. Second, the dominant narrative that links Islamist insurgents to polio is associated with Western commentators, many of whom see – implicitly or explicitly – Islamist insurgents as enemies of Western interests. It is possible that this narrative is “system-supportive propaganda” aimed at disparaging Islamist insurgents and legitimizing military intervention [30].

Our dataset includes all countries with a population >100,000 and covers the period 2003, the year of the Nigerian boycott, to 2014. The dependent variable is the number of wild polio cases as enumerated by the WHO [3]. In the regressions we use the 3-year mean of polio cases in a country. This flattens out year-to-year fluctuations. It also better operationalizes the proposed mechanism that links insurgency and polio: insurgency and/or insurgents disrupt vaccination programmes, not enough children are vaccinated, and this may lead to an outbreak in the same year or subsequent years.

To code the key independent variable – whether or not a country is affected by insurgency – we use the Uppsala Conflict Data Programme *Armed Conflict Dataset* (ACD) [31]. Civil War is defined as a conflict between the state and opposition groups in which there were 25 or more battle deaths in a calendar year. The ACD lists the main opposition groups in each conflict but we code whether they are Islamist or not ourselves. We define Islamist insurgents as groups that use Islam to legitimize their activities and are seeking to overthrow the state and replace it with an Islamist state. All other opposition groups are coded as non-Islamist insurgents. For more information on the coding of insurgency see the Additional file 1.

**Fig. 1:**
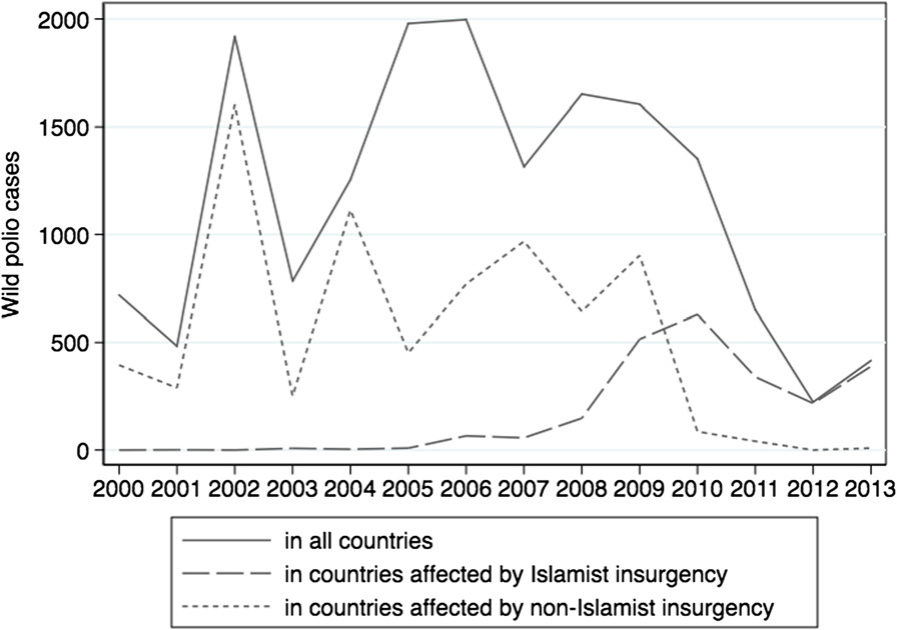
Insurgency and polio cases, 2000-13

Figure 1 illustrates the proportion of polio cases that occurred in countries affected by Islamist and non-Islamist insurgency between 2000 and 2013. In the early-2000s the vast majority of polio cases occurred in countries that were affected by non-Islamist insurgency. There was a decline in polio cases in countries affected by non-Islamist insurgency throughout the period to virtually none by 2010. Conversely, until 2008 countries affected by Islamist insurgency accounted for a very small proportion polio cases. From 2008 the total number of polio cases in the world began to fall sharply. Concomitantly, the number of polio cases occurring in countries affected by Islamist insurgents increased markedly. By 2010 the majority of polio cases occurred in countries affected by Islamist insurgency.

We use negative binomial regression to test and quantify the relationship between insurgency – both Islamist and non-Islamist – and polio. We choose negative binomial regressions because our dependent variable is count data and the conditional distribution of the dependent variable is over-dispersed, making Poisson regression unsuitable [32]. We are primarily interested in cross-sectional regressions because we want to investigate whether the determinants of polio change over time. Nevertheless we present the results of time-series analyses in the Additional file 1.

We control for other possible explanatory variables in order to test whether the relationship between insurgency and polio is spurious. First, we control for per capita GDP as both insurgency [28] and disease [29] are more likely to occur in less developed countries. Second, countries with large populations are *ceteris paribus* more likely to experience more polio cases and insurgencies. We therefore control for the total population. Third, we control for the proportion of the total population that live in rural areas because insurgency is more likely to occur in rural areas where the state has less of a presence [28] and it is more difficult for polio immunization workers to reach rural areas where transport and health infrastructure are often poor [8, 9]. Fourth, as polio is more likely to occur among populations where the general level of health is worse we control for the Infant Mortality Rate. All these variables are coded from World Development Indicators. Fifth, as polio is infectious, a country is more likely to be affected by polio if it was affected by polio in the previous year. We add a variable for the number of wild polio cases a country experienced in the previous year. The population and previous polio cases variables are log transformed to make their distribution less skewed.

Table 1 shows descriptive statistics for the main variables.

**Table 1:** Overview of main variables

	Observations	Mean	Standard deviation	Minimum	Maximum
Polio cases (absolute)	1920	6.471	58.058	0	1122
Polio cases (3 year mean)	1920	6.387	50.153	0	911.333
Islamist insurgency	1920	.030	.171	0	1
All Insurgency	1920	.108	.311	0	1
Non-Islamist insurgency	1920	.139	.346	0	1
GDP per capita (000)	1895	11.648	17.504	.108	113.739
Total population (log)	1920	15.639	1.928	11.378	21.060
Infant Mortality Rate	1910	31.894	28.738	1.7	134.4
Rural population %	1820	44.664	23.672	0	91.077
Polio in previous year (log)	1920	.239	.931	0	7.378

We all include all countries with a population > 100,000 for the period 2003–2012

## Results and discussion

In this section we set out the results of the negative binomial regressions. We report incidence rate ratios (IRRs), the exponentiated regression coefficient. Robust standard errors are in parentheses.

In Table 2 the data for the independent variables are from 2003 – when Islamists in Nigeria first advocated a boycott of polio vaccination programmes – and the dependent variable is the mean annual number of polio cases in the period 2003–05. There was a significant negative relationship between Islamist insurgency and polio (models 1,3,5,7). In model 7, when we control for other explanatory variables, countries affected by Islamist insurgency had five times fewer polio cases than those that were not (*p* = .019). In model 3 there is a positive significant relationship between non-Islamist insurgency and polio (IRR = 6.618, *p* = .049). But non-Islamist insurgency loses significance when we control for other possible explanatory variables (model 7). This suggests that the relationship between non-Islamist insurgency and polio is spurious: it is not insurgency, but variables that increase the likelihood of insurgency such as a large population and low level of development, that increase country’s susceptibility to polio. Of the control variables, total population and infant mortality rate have a significant positive relationship with polio (models 4–7).

**Table 2:** Negative binomial regression testing the relationship between Islamist insurgency in 2003 and polio cases in 2003–05 (3-year mean)

	1	2	3	4	5	6	7
Islamist insurgency	.198^**^ (.094)		.242^*^ (.133)		.192^*^ (.143)		.194^*^ (.136)
Insurgency		5.720 (5.460)				.533 (.533)	
Non-Islamist insurgency			6.618^*^ (6.342)				.601 (.635)
GDP per capita (000)				.875 (.124)	.871 (.133)	.854 (.151)	.856 (.150)
Total population (log)				4.223^***^ (1.177)	4.086^***^ (1.133)	5.003^***^ (2.013)	4.730^***^ (2.001)
Infant Mortality Rate				1.056^***^ (.016)	1.054^**^ **(**.016)	1.059^***^ (.016)	1.057^***^ (.016)
Rural population %				1.051 (.032)	1.054 (.033)	1.050 (.030)	1.052 (.030)
Previous polio	2.746^***^ (1.071)	3.100^**^ (1.118)	3.274^**^ (1.281)	.932 (229)	.972 (.243)	.841 (.258)	.885 (.296)
Observations	192	192	192	182	182	182	182

We report incidence rate ratios. Standard errors are in parentheses. Constants are calculated but not reported

^*^ *p* < .05 (5 %), ^**^ *p* < .01 (1 %), ^***^ *p* < .001 (0.1 %)

In Table 3 the data for the independent variables are from 2012 and the dependent variable covers 2012–2014. Model 8 demonstrates that, when we do not control for other possible explanatory variables, a country that was affected by Islamist insurgency in 2012 will have 110 times more polio cases than a country that is not affected by Islamist insurgency (*p* < .001). In model 9 a country that was affected by any type insurgency in 2012 will have 49 times more polio cases (*p* < .001). In model 10 we disaggregate the key independent variable: only Islamist insurgency is significant. In model 12, when we control for other possible explanatory variables, the IRR for Islamist insurgency falls but remains significant at the .1 % level (IRR = 30). In model 13 all insurgency is significant (*p* < .001) but the IRR (12.5) is smaller than for Islamist insurgency. This suggests that Islamist insurgency in particular, rather than insurgency in general, is driving the relationship. This is confirmed when we disaggregate insurgency into two dummy variables in model 14. Islamist insurgency is significant (IRR = 28, *p* < .001) but non-Islamist insurgency is not significant. Of the control variables, total population and infant mortality rate have a significant positive relationship with polio (models 10–14).

**Table 3:** Negative binomial regression testing the relationship between Islamist insurgency in 2012 and polio cases in 2012–14 (3-year mean)

	8	9	10	11	12	13	14
Islamist insurgency	110.078^***^ (96.727)		126.742^***^ (112.982)		29.695^***^ (18.510)		27.889^***^ (17.521)
Insurgency		48.686^***^ (41.103)				12.503^***^ (7.829)	
Non-Islamist insurgency			2.766 (2.966)				.659 (.754)
GDP per capita (000)				.394^*^ (.162)	.588^*^ (.119)	523^***^ (095)	.582^*^ (.112)
Total population (log)				3.329^***^ (1.014)	2.528^**^ (.727)	2.572^**^ (795)	2.589^***^ (.693)
Infant Mortality Rate				1.045 (.027)	1.026 (.014)	1.032 (.018)	1.026^§^ (.025)
Rural population %				.920^*^ (.032)	.967 (.024)	.935^*^ (.025)	.969 (.025)
Previous polio	2.525^***^ (.614)	2.573^***^ (.557)	2.580^***^ (.659)	1.343 (.293)	1.445 (.281)	1.486^*^ (.269)	1.417 (.282)
Observations	192	192	192	183	183	183	183

We report incidence rate ratios. Standard errors are in parentheses. Constants are calculated but not reported

^*^ *p* < .05 (5 %), ^**^ *p* < .01 (1 %), ^***^ *p* < .001 (0.1 %), §*p* = .05 (5 %)

In Table 4 we replicate models 7 and 14 for the years between 2003–5 and 2012–14 in order to better understand how the relationship between insurgency and polio has changed over time. Several trends can be observed. First, there was significant negative relationship between Islamist insurgency and polio at the start of the period (model 15), no significant relationship with polio throughout most of the period, but a significant positive relationship with insurgency for the last 3 years of the period (models 22–24). It is interesting to note that the first point at which there was a significant positive relationship between Islamist insurgency and polio was the first time that the dependent variable included polio cases that occurred after the assassination of bin Laden in 2011. Second, non-Islamist insurgency is significant at the 5 % level in two periods – in 2006–08 and 2007–09 (models 18–19). Nevertheless, it seems likely that this relationship is the result of an ecological fallacy. In the period 2006–09 India accounted for 70 % of polio cases in countries affected by non-Islamist insurgencies. But there does not appear to be a causal relationship between insurgencies – which occurred in Kashmir, the north-east and the central tribal belt – and polio, which was most prevalent in Uttar Pradesh and Bihar [33, 34]. The relationship between Islamist insurgency and polio does not appear to be the result of an ecological fallacy (see Additional file 1).

**Table 4:** Negative binomial regression testing the relationship between Islamist insurgency and polio (3 year mean), 2003–2014

	15	16	17	18	19	20	21	22	23	24
	2003–5	2004–6	2005–7	2006–8	2007–9	2008–10	2009–11	2010–12	2011–13	2012–14
Islamist insurgency	.194^*^ (.136)	.218 (.197)	.305 (.226)	1.596 (1.130)	1.156 (.586)	.335 (.271)	.183 (.203)	49.313^***^ (50.731)	10.359^**^ (8.454)	27.889^***^ (17.521)
Non-Islamist insurgency	.601 (635)	1.291 (1.429)	.392 (.289)	2.867^*^ (1.114)	4.895^*^ (3.073)	.436 (.440)	.430 (.512)	2.634 (1.522)	1.058 (.732)	.659 (.754)
GDP per capita (000)	.856 (.150)	.949 (.039)	.918 (.049)	.990 (.026)	.311^**^ (.121)	.927^*^ (.061)	.807 (.106)	.878 (.062)	.730^*^ (.113)	.582^*^ (.112)
Total population (log)	4.730^***^ (2.001)	3.229^*^ (1.544)	4.066^***^ (1.619)	1.733 (.594)	2.478^***^ (.619)	1.936^**^ (.402)	1.486^*^ (.265)	2.065^**^ (.446)	2.227^*^ (.686)	2.589^***^ (.693)
Infant mortality (per 1000)	1.057^***^ (.016)	1.049^**^ (.017)	1.043^*^ (.019)	1.036^*^ (.015)	1.059^***^ (.013)	1.042^***^ (.008)	1.072^***^ (.019)	1.103^***^ (.024)	1.044^**^ (.017)	1.026§ (.025)
Rural population %	1.052 (.030)	1.058^*^ (.028)	1.061^*^ (.030)	1.034^**^ (.011)	.975 (.021)	1.055 (.031)	.990 (.028)	.938^*^ (.159)	.960 (.021)	.969 (.025)
Polio cases in previous year (log)	.885 (.296)	1.025 (.234)	1.036 (.183)	2.100^***^ (.381)	1.332 (.211)	1.417^*^ (.246)	1.450 (.373)	.743^*^ (.159)	2.116^***^ (.387)	1.417 (.282)
Observations	182	182	182	182	182	183	183	183	183	183

We report incidence rate ratios. Standard errors are in parentheses. Constants are calculated but not reported

^*^ *p* < .05 (5 %), ^**^ *p* < .01 (1 %), ^***^ *p* < .001 (0.1 %), ^§^ *p* = .05 (5 %)

Third, in all regressions there is a positive significant relationship between total population and the number of polio cases (models 15–24). This reflects the fact that, *ceteris paribus*, countries with larger populations are likely to have more polio cases. Fourth, in all the regressions there is a positive significant relationship between infant mortality – a proxy for the general health of the population – and the number of polio cases (models 15–24). If a country’s Infant Mortality Rate (deaths per 1000 live births) decreased by one it would expect to see a fall in the number of polio cases of between 3 and 10 % depending on the year when all other variables are held constant. Fifth, there is a significant positive relationship between the proportion of the population living in rural areas and the polio at the beginning of the period under analysis (models 16–18). Model 15 is close to being significant at the 5 % level (*p* = .077). But rural population is not significant in later periods with the exception of model 22 where there is a significant negative relationship. It seems likely that recent improvements in vaccines and vaccine programmes have allowed undermined the challenges the rural locations posed to eradication.

We performed a variety of robustness tests on the results presented in this section. First, we ran the regressions with an alternative operationalization of Islamist insurgency that uses a threshold of more than 1000 deaths per year. The results were similar, demonstrating that our analysis is robust to different definitions of insurgency. Second, we included additional control variables. For example, we reran the regressions for the periods in which Islamist insurgency had a positive significant relationship with polio with a variable for the log population of Muslims in a country. The variable was significant in all models when Islamist insurgency was not included. But when Islamist insurgency is included the proportion of Muslims loses significance. This reflects the fact that Islamist insurgents’ attitudes towards polio should be understood in the context of Islamists’ interaction with domestic political rivals and international actors. Third, we set out qualitative data to demonstrate that the relationship between Islamist insurgency and polio is not the result of an ecological fallacy. The robustness tests do not alter our main conclusions. For more details of the robustness tests see the Additional file 1.

## Discussions and conclusions

The main barriers to polio eradication are no longer medical or technical because of improvements in vaccines and the administration of vaccines. Polio persists and continues to spread because of political and social barriers that stop the effective implementation of polio eradication programmes. This paper analysed cross-national data to investigate the relationship between different forms of insurgency and polio between 2003 and 2014. Islamist insurgency did not have a significant positive relationship with polio throughout the whole period. But in the past few years Islamist insurgency has had a strong effect on where polio cases occur. This is because Islamist insurgents deliberately undermine the efficacy of immunization campaigns by spreading misinformation and attacking polio workers. The evidence for all types of non-Islamist insurgency is less compelling. First, in some cases the relationship is no longer significant when we control for variables that operationalize the level of development in a country. This suggests that the relationship is spurious. Second, in a few cases non-Islamist insurgency was significantly related to polio even when we controlled for other explanatory variables but this relationship was the result of an ecological fallacy. This challenges the view that armed conflict in general is driving the persistence and re-emergence of polio in some areas [1, 6, 12, 13]. Notwithstanding this, it makes sense as, to the best of our knowledge, there is no evidence that non-Islamist insurgents intentionally aim to disrupt polio eradication programmes. Left wing insurgents, such as the Communist Party of India (Maoist), aim to generate mass support by improving public health in areas under their control and do not harm polio workers [35]. On the other hand, insurgents that have narrower political or economic objectives have little interest in generating support through social medicine but are not opposed to vaccination campaigns.

Our analysis supports the position that Islamist insurgents’ hostility towards polio vaccination programmes negatively affects the implementation of the GPEI. This is not to say that we advocate a renewed “War on Terror” to complement the war on polio. The negative consequences of military intervention would far outweigh the possible public health gains – even if it precipitated the eradication of polio. The question of why Islamist insurgents are hostile to polio vaccination programmes can only be answered definitively with qualitative research. Nevertheless, our findings contribute some evidence to this debate. If Islamist insurgents’ antagonism to polio vaccinations was driven by their theology we would expect it to be constant throughout the period studied because the basic theological tenets of Islam have remained more or less constant. This is not the case. We noted above that Islamists are not inevitably hostile to polio eradication campaigns. For example, when the Afghani Taliban were in power they fully supported the GPEI and even al-Qaeda did not attempt to disrupt it [9], and Fatwas issued against polio workers in Pakistan have led to counter fatwas by other religious authorities urging vaccination [23]. It seems apparent that some Islamist insurgents have come to realize that targeted violence and boycotts against polio programmes is a useful strategy: it can be used to generate international attention for the insurgents and to force concessions from their opponents. What is more, it appears to be that case that some Islamist insurgents have become increasingly hostile towards polio vaccination campaigns as a result of the counterinsurgency strategies of domestic governments and international actors. The increased use of drone attacks and the CIA’s use of a fake immunisation program in the assassination of Osama bin Laden have seemingly vindicated Islamist insurgents’ suspicions that immunization drives are a cover for espionage activities and seriously compromised the GPEI’s activities. These are important lessons for Western policy makers, international organizations, and governments in the Middle East and North Africa as they deliberate how best to counter the growing influence of Islamist insurgents.

## Additional file

Additional file 1:
**Appendix.** (DOCX 114 kb)
