# Supplementary material for: Islamist insurgency and the war against polio: a cross-national analysis of the political determinants of polio
Source: Global Health. 2015 Sep 30;11:40. doi: 10.1186/s12992-015-0123-y (PMC4589183; doi:10.1186/s12992-015-0123-y)
Supplement: Additional file 1: — Appendix. (DOCX 114 kb) [file 12992_2015_123_MOESM1_ESM.docx]

APPENDIX

*Notes on Coding*

Our operationalization of insurgency includes what the Uppsala Conflict Data Programme *Armed Conflict Dataset (*ACD) refers to ‘internal armed conflict’ between the state and one or more internal opposition groups, as well as ‘internationalized internal armed conflict’ in which external states intervene on one or both sides[1]. The ACD codes two major forms of incompatibility: government and territory. Insurgents whose main incompatibility is government aim to seize the whole territory of a state. Islamist insurgent groups whose main incompatibility is government include Afghani and Pakistani Taliban, Islamic State, Boko Haram, al-Shabaab and al-Qaeda affiliates in North Africa and the Middle East. We code these insurgencies as Islamist insurgencies. Insurgent organizations whose main incompatibility is territory are fighting for the independence or autonomy of a particular region in a state. Some insurgent organizations involved in territorial conflicts legitimize their activities with reference to Islam, but the underlying incompatibility concerns a spatially concentrated Muslim minority’s desire for independence or autonomy from non-Muslim majority countries. For this reason we do not code territorial conflicts as Islamist insurgencies. Such groups include Kashmiri, Palestinian and Chechen insurgents in India, Israel/Palestine, and Russia respectively. The ACD dataset codes the United States’ as being affected by an armed conflict with al-Qaeda, but we do not include this in our list of Islamist insurgencies because al-Qaida does not primarily aim to overthrow the American state and replace it with an Islamist state.

*Time series analysis*

In the main body of the paper we analyse cross-sectional data because we are primarily interested in changes in the determinants of polio over time. Notwithstanding, we analysed our dataset as panel data for the years 2003-14, using negative binominal regression with fixed effects for country and year. Count data fixed effects models drop groups in which all outcomes are zero. As a result we lose 138 country groups and 1375 observations in which there were no polio cases in the 10 years from 2003. We are left with 44 country groups and 440 observations. The countries that remain are overwhelmingly developing countries in Africa and Asia. The extent of variation in our sample is obviously lower in the time series analysis in comparison to the cross-sectional analysis. We make one change to the variables we include in the time-series models. We drop the variable for whether polio occurred in the previous year because of collinearity. This is not a problem because country fixed effects will control for the fact that a country is more likely to be affected by polio if it was affected by polio in the previous year.

[INSERT TABLE A1 HERE]

The results of negative binomial regression with country and year fixed effects are set out in table A1. As in the tables in the main body of the paper we report incidence rate ratios (IRR), the exponentiated regression coefficient. Robust standard errors are in parentheses. The results are as we might expect from looking at the cross-sectional analysis.

Islamist insurgency is only significant at the 5% level in one model (model A3). But in model A1 when Islamist insurgency is included on its own (p=.059) and model A7, when we include control variables (p=.065), it is close to being significant at the 5% level. This suggests that there is a relationship between Islamist insurgency and polio. Nevertheless, the time-series analysis conceals the manner in which the relationship between Islamist insurgency and polio has developed over the past 10 years. In the main body of the paper we present a variety of data to show that the relationship between Islamist insurgency and polio is a recent phenomenon.

All insurgency is significant in model A2 at the 0.1% level and almost significant in model A6 when we control for other explanatory variables (p=.063). But when we disaggregate all insurgency it is apparent that Islamist insurgency in particular, rather than insurgency in general, is driving the relationship with polio (models A3 and A7).

Non-Islamist insurgency is significant at the 0.1% level when we include it in the regression without controls (model A3). But non-Islamist insurgency (model A7) loses significance when we control for other possible explanatory variables (model 7). This provides further evidence that the relationship between non-Islamist insurgency and polio is spurious: it is not insurgency, but variables that increase the likelihood of insurgency, such as a large population and low level of development, that increase the probability that a country will experience polio.

Of the control variables, three have a significant positive relationship with polio: total population and infant mortality rate were consistently significant in the cross-sectional analysis, while the rural population was not (models A4 to A7). First, total population reflects the fact that, *ceteris paribus*, countries with larger populations are likely to have more polio cases. Second, infant mortality captures the general level of public health in a country. If a country’s Infant Mortality Rate (deaths per 1,000 live births) decreased by one, we would expect to see a fall in the number of polio cases of about 4%, when all other variables are held constant. Third, the time series analysis suggests that countries with a higher proportion of the population living in rural areas are likely to have a higher number of polio cases. This is because it is more difficult for polio immunization workers to reach rural areas where transport and health infrastructure are often poor. Table A1 suggests that a 1% decrease in the rural population would lead to a 3.5% fall in the number of polio cases when all other variables are held constant. The cross-sectional analysis indicates that this relationship is driven by observations in the early part of the period that we analysing. It seems likely that recent improvements in vaccines and vaccine programmes have allowed the GPEI to overcome the challenges posed by rural locations.

*Robustness tests*

We carried out a number of robustness tests on the results reported in tables 2,3 and 4 in the main text. These do not alter our conclusions.

First, we use an alternative operationalization of Islamist insurgency. The ACD defines internal armed conflict as a conflict in which there were 25 or more battle deaths in a calendar year. Although this definition is more or less ubiquitous in quantitative analyses of civil war, the threshold of 25 is arbitrary. We therefore test whether our regression results are robust to insurgency being defined as an armed conflict resulting in more than 1,000 deaths per year – the alternative threshold coded by the ACD. This substantially reduces the number of insurgencies each year – for example, in 2012 there were 31 internal armed conflicts with ≥25 battles deaths but only six when the threshold is increased to 1,000. Nevertheless, we get similar results when we rerun the regressions in tables 2, 3 and 4 with the alternative definition, demonstrating that our analysis is robust to different definitions of insurgency.

Second, in tables 2, 3 and 4 we control for the total population because countries with large populations are *ceteris paribus* more likely to experience more polio cases. Ordinarily rates would be used to address this issue but the number of cases is so small that this is not appropriate. It could be argued that, given one case is one case too many, there is no need to control for population. We therefore run the regressions without the population control. This does not alter our main conclusions.

Third, the regressions in the main paper use infant mortality rate as a control for the general level of health in a country. Infant mortality rate is significant in the vast majority of models in our analysis. Even where it is not significant at the 5% level (i.e., in table 3) it is close to the 5% level. While there is considerable overlap, maternal mortality tends to be a measure of healthcare whereas infant mortality better captures the general level of public health in a country. These variables are too highly correlated to be included in the same regression model (r=0.914, p<.001). We reran the regressions with maternal mortality rate as an alternative control to infant mortality. Maternal mortality is significant in all models except for 2009 to 2011 and 2008 to 2010. As a result of the strong correlation between infant mortality and maternal mortality it is difficult to discern whether it is the level of health, the level of healthcare, or a combination of the two that increases the likelihood of polio.

Fourth, an alternative explanation is that “misinformed and politicised religious views” associated with Islam rather than Islamist insurgency explains the global distribution of polio[2,3]. Such a view is supported by the fact that in India poliovirus was most persistent among Muslim communities in Uttar Pradesh and Bihar where there was no Islamist insurgency but where rumours about vaccinations were rife[4]. To test the possible relationship between Islam and polio we reran the regressions for the periods in which Islamist insurgency had a positive significant relationship with polio, including a control variable for the log population of Muslims in a country according to the Pew Research Centre[5]. This variable is significant in all models when Islamist insurgency is not included in the regression. But when Islamist insurgency is included in the regression the proportion of Muslims loses significance. This reflects the fact that Islamist insurgents’ attitudes towards polio should not be understood with reference to Islam but in the context of their interaction with domestic political rivals and international actors. Indeed, it should be noted that not all Muslims are hostile towards polio vaccination. For example, a group of the world’s leading Islamic scholars led by the Grand Imam of the Holy Mosque of Mecca issued the Jeddah Declaration in February 2014, which stated that “protection against diseases is obligatory and admissible under Islamic Shariah, and that any actions which do not support these preventive measures and cause harm to humanity are un-Islamic”[6-7].

Fifth, it is possible that there may be an ecological fallacy in our results. Both Islamist insurgencies and polo outbreaks tend to be concentrated in specific regions of a country. It is plausible that these regions could be different. This would not be picked up in national level data. Nevertheless, qualitative evidence suggests that Islamist insurgencies and polo outbreaks occur in the same parts of affected countries[8]. Polio outbreaks are concentrated in northern Nigeria, the stronghold of Boko Haram[9]; in Pakistan, polio cases are concentrated in the Federally Administered Tribal Areas, where the Taliban is strongest[9]; polio in Afghanistan is concentrated in areas over the border from insurgent-affected areas in Pakistan[9]; in Somalia polio occurs in the South, which is al-Shabaab’s powerbase[10]; and polio in Syria is concentrated in and around Islamist insurgent held areas of Aleppo and Deir al-Zour[11]. On the other hand, the relationship between non-Islamist insurgency and polio appears to be the result of an ecological fallacy. In India poliovirus was most persistent among Muslim communities in Uttar Pradesh and Bihar. India is affected by several civil wars – in Kashmir, in the north-east and in the central tribal belt – but none of these involved the Muslim communities of Uttar Pradesh and Bihar[4,12] .

APPENDIX REFERENCES

1. Uppsala Conflict Data Programme (undated). Armed Conflict Dataset. <http://www.pcr.uu.se/research/ucdp/datasets/ucdp_prio_armed_conflict_dataset/>

2. Ahmed, Q. A., Nishtar, S., and Memish, Z. A. (2013). Poliomyelitis in Pakistan: time for the Muslim world to step in. *Lancet*, *381* (9877), 1521-1523.

3. Kaufmann, J. R., and Feldbaum, H. (2009). Diplomacy and the polio immunization boycott in Northern Nigeria. *Health Affairs* *28* (4), 1091-1101.

4. Pallansch, M. A., and Sandhu H. S. (2006). The eradication of polio—progress and challenges. *New England Journal of Medicine* 355 (24), 2508-2511.

5. GPEI. (2014). Islamic scholars call for access to vaccinate children <http://polioeradication.org/tabid/488/iid/355/Default.aspx>

6. Pew Research Centre. (Undated). Muslim Population by Country <http://www.pewforum.org/2011/01/27/table-muslim-population-by-country/>

7. Riaz, F., Waheed, Y. (2014). Islam and polio. *Lancet Infect Dis*, 14 (9):79179-2.

8. Kennedy, J.J., (undated). The political determinants of polio: A comparative historical analysis of the relationship between Islamist insurgency and polio in Nigeria, Somalia, Pakistan, Afghanistan and Syria. Unpublished Manuscript

9. Abimbola, S., Malik, A. U., and Mansoor, G. F. (2013). The final push for polio eradication: addressing the challenge of violence in Afghanistan, Pakistan, and Nigeria. *PLoS medicine*, *10* (10), e1001529.

10. Burki, T. K. (2013). Somalia: a gathering storm?. *Lancet* 382 (9900), 1237-1238.

11. Sparrow, A. (2014). Syria’s Polio Epidemic: The Suppressed Truth. *New York Review of Books* (20^th^ February).

12. Kennedy, J.J., & King, L.P. (2011). Understanding the conviction of Binayak Sen: Neocolonialism, political violence and the political economy of health in the central Indian tribal belt. *Social Science & Medicine*, *72* (10), 1639-1642.

| **Table A1:** Negative binomial regression with fixed effects for country and time for period 2003-2013 | | | | | | | |
| --- | --- | --- | --- | --- | --- | --- | --- |
|  | 1 | 2 | 3 | 4 | 5 | 6 | 7 |
| Islamist insurgency | 1.643§ (.432) |  | 2.111* (.583) |  | 1.520 (406) |  | 1.668§ (.461) |
| Insurgency |  | 2.155*** (.408) |  |  |  | 1.442§ (.283) |  |
| Non-Islamist insurgency |  |  | 2.176*** (.459) |  |  |  | 1.338 (.300) |
| GDP per capita (000) |  |  |  | .961 (.099) | .958 (.100) | .963 (.099) | .961 (.099) |
| Total population (log) |  |  |  | 1.596*** (.133) | 1.615*** (.136) | 1.527*** (.132) | 1.551*** (.139) |
| Infant Mortality Rate |  |  |  | 1.038*** (.005) | 1.038*** (.005) | 1.038*** (.005) | 1.038*** (.005) |
| Rural population (%) |  |  |  | 1.037** (.012) | 1.037** (.012) | 1.034* (.012) | 1.035* (.013) |
| Observations | 440 | 440 | 440 | 440 | 440 | 440 | 440 |
| Notes:- We report incidence rate ratios. Standard errors are in parentheses. Constants are calculated but not reported. * = p <.05 (5%), ** = p <.01 (1%), *** = p <.001 (0.1%), § = p < .07 (7%) | | | | | | | |
